# Immunoassays and Biosensors for the Detection of Cyanobacterial Toxins in Water

**DOI:** 10.3390/s131115085

**Published:** 2013-11-05

**Authors:** Michael G. Weller

**Affiliations:** Division 1.5 Protein Analysis, BAM Federal Institute for Materials Research and Testing, Richard-Willstätter-Strasse 11, 12489 Berlin, Germany; E-Mail: michael.weller@bam.de; Tel.: +49-30-8104-1150; Fax: +49-30-8104-1157

**Keywords:** algal toxins, cyanotoxins, biotoxins, blue-green algae, harmful algal blooms, microcystins, nodularins, anatoxin-a, anatoxin-a(s), cylindrospermopsin, saxitoxins

## Abstract

Algal blooms are a frequent phenomenon in nearly all kinds of fresh water. Global warming and eutrophication by waste water, air pollution and fertilizers seem to lead to an increased frequency of occurrence. Many cyanobacteria produce hazardous and quite persistent toxins, which can contaminate the respective water bodies. This may limit the use of the raw water for many purposes. The purification of the contaminated water might be quite costly, which makes a continuous and large scale treatment economically unfeasible in many cases. Due to the obvious risks of algal toxins, an online or mobile detection method would be highly desirable. Several biosensor systems have been presented in the literature for this purpose. In this review, their mode of operation, performance and general suitability for the intended purpose will be described and critically discussed. Finally, an outlook on current developments and future prospects will be given.

## 1. Introduction

The occurrence of algal blooms (the mentioned species belong to the division of cyanobacteria; formerly, they had been designated as blue-green algae) has been reported since ancient times from nearly all parts of the World. One of the best known supposed references is from the Bible describing the ten plagues. The first one was the plague of blood (Exodus 7:17–18): “… *I will strike the water of the Nile, and it will be changed into blood. The fish in the Nile will die, and the river will stink and thus the Egyptians will not be able to drink its water*.” This report already describes two of the known major risks of toxic algae, the threat to the fishery and to drinking water production. In more recent times, the blooms in the Lake Zurich, Switzerland, have been described repeatedly. The cyanobacterium *Planktothrix rubescens* [1] is a producer of microcystins, an important hepatotoxic compound. Microcystins (http://en.wikipedia.org/wiki/Microcystin) are cyclic heptapeptides with varying amino acids, which lead to a specific nomenclature [2]. Nodularins (http://en.wikipedia.org/wiki/Nodularin) are pentapeptides of a similar structure produced by *Nodularia spumigena*, regularly forming blooms in the Baltic Sea, which is a brackish habitat [3]. Microcystins are produced by the eponymous *Microcystis* species, which belong to the most relevant toxin-producers in fresh-water. Both compounds, microcystins and nodularins, are hepatotoxins and may kill animals or humans by causing severe liver damage. Other cyanotoxins belong to the group of alkaloids, such as anatoxin-a, anatoxin-a(s), cylindrospermopsin, saxitoxin and others. More information about cyanobacteria and cyanobacterial toxins can be found, e.g., on the well-known Cyanosite [4]. In addition, some interesting reviews have been published [5–10]. This paper is not focused on marine biotoxins [11], however, some antibodies or biosensors are briefly mentioned.

The analysis of cyanobacterial toxins is quite demanding. The group of the microcystins alone consists of more than 70 variants (“congeners”) [12], from which only a few are available as standard substances. Although microcystins and the related nodularins both contain the very specific amino acid (2*S*,3*S*,8*S*,9*S*)-3-amino-9-methoxy-2,6,8-trimethyl-10-phenyldeca-4,6-dienoic acid (Adda), with a UV absorbance maximum around 238 nm, complex samples obtained from algal blooms and contaminated waters often do not allow the unambiguous determination of all microcystins at a trace level. Even LC-MS/MS, which is usually a very powerful method used for environmental analysis, may fail here, since neither the retention times of all toxins can be determined, nor the molecular masses (or transitions) of all potential toxins are known. Therefore, more sophisticated approaches are needed, such as the use of Adda-selective antibodies, which are able, at least in theory, to identify all possible congeners of microcystins and nodularins (and even other unknown Adda-containing toxins). Except for immunoassays, which are commercially available, the most frequently used analytical technique is solid-phase extraction (SPE) combined with HPLC and UV detection. The use of mass spectrometry as a detector becomes more and more popular, since the identification of the toxins is more reliable, particularly, if high-resolution mass-spectrometry (HRMS) is used. The quantitation is still difficult, since no isotopically labeled standards are available for the application of isotope dilution techniques. The quantitation via UV absorbance can be adequate, as long as the toxin level is high enough, the matrix does not interfere and the toxin peaks can be properly assigned. Unfortunately, interferences are frequent in the respective UV range. The threshold value of microcystin-LR for drinking water proposed by the World Health Organization (WHO; http://www.who.int/en/) can be monitored by a standardized HPLC method issued by the DIN/ISO in 2005 and 2007 [13]. Most HPLC methods are variants of a method published by Lawton *et al.* in 1994 [14]. Meriluoto published a review about the chromatography of microcystins some years later [15]. One big problem of microcystin and nodularin analysis is not only the undefined number of chemical species, which have to be considered, but also their highly variable toxicity. This review will not cover details regarding the toxicities of the different congeners; only two major papers should be mentioned here [16,17]. However, it has to be stressed that as long as the analysis of a sample has the aim to identify a “toxic hazard”, the application of an approach known as effect-directed analysis is highly recommended [18], as otherwise an analytically precise, but misleading conclusion might result. One concept of this kind was shown by Zeck *et al.* [19] by the combination of protein phosphatase inhibition (toxicity related), antibody inhibition (structure related), and UV absorbance at 238 nm (quantity). In addition, it is proposed that parallel mass-spectrometric analysis might be used for confirmation of known microcystins. As one example of a severe cyanobacterial intoxication, the Caruaru case (Brazil) should be mentioned [20,21], where in a hemodialysis unit more than 60 patients died, most probably due to a microcystin contamination of the water. In this context, the usefulness of rapid biosensors for cyanobacterial toxins might become obvious. Highly time-dependent hazards in connection with severe algal bloom events are typical for cyanobacterial toxins. Therefore, occasional sampling with analytical examination in the laboratory is not adequate under real conditions. Heavy toxin peaks might be missed completely, and hence the laboratory results would be of not much advantage, as long as very short sampling intervals cannot be realized due to cost, accessibility or other limitations. Furthermore, the time delay between sampling and availability of the final analytical result might be too long, particularly in remote sampling areas considering typical drinking water production units, in which the raw water is nearly immediately processed to drinking water and delivered to the customers.

In contrast to microcystins and nodularins, the analysis of saxitoxins (http://en.wikipedia.org/wiki/Saxitoxin) [22], anatoxin-a (http://en.wikipedia.org/wiki/Anatoxin-a) and anatoxin-a(s) (http://en.wikipedia.org/wiki/File:Anatoxin-a-S.png) [23–27] often require non-standard analytical techniques, such as capillary electrophoresis or chemical derivatization in combination with chromatographic techniques. In contrast, cylindrospermopsin (http://en.wikipedia.org/wiki/Cylindrospermopsin) [28–31] may be analyzed by usual HPLC/UV or LC-MS/MS, like the microcystins. Some reviews have been already published [32–34], which cover some aspects of this article. The reader is encouraged to take a look at these articles, too.

## 2. Immunoassays

Immunochemical and other biochemical methods always have been important for the detection of cyanobacterial toxins [35]. Commercial immunoassay kits and antibodies are available for at least some cyanotoxins. In Tables 1 and 2 (not exhaustive—the most recent information should be requested from the corresponding manufacturers) some commercial tests are listed with their major performance characteristics.

Many immunoassays have been reported in the literature, which are not available on the market. Some of the respective antibodies might even been lost in between—a general problem of immunoanalytical techniques, which has not been resolved in many fields. In addition, often it is not easy to specify the antibodies used in a respective immunoassay system. Considering the fundamental influence of the antibody on assay performance, this is a significant drawback of some tests. It occurred not only once that assay developers switched to different antibodies for the manufacturing of an immunoassay, often without any notice to the customer.

One of the first polyclonal (rabbit) antibodies against microcystins was reported by Brooks and Codd in 1988 [38] and by Chu, Huang, Wei and Carmichael in 1989 [39]. Antibodies from chicken had been developed by McDermott, Feola and Plude [40]. Metcalf *et al.* presented a now popular method for the conjugation of microcystins to proteins, based on a derivatization with 2-mercapto-ethylamine [41]. Sheng *et al.* produced polyclonal antibodies based on a microcystin-LR conjugate, which displayed a quite similar cross-reactivity for a series of microcystins and nodularin [42]. Young *et al.* raised polyclonal antibodies (rabbit) against microcystin-LR and microcystin-RR [43]. Surprisingly, the resulting cross-reactivities were not very different. This might be an indication of the relative immunodominance of Adda, which is part of the conserved area of microcystins. Baier *et al.*, tried the use of polylysine microcystin conjugates in combination with lipopeptide adjuvants [44], however, with limited success.

The first monoclonal antibodies (IgM) against microcystins (the cyanoginosins mentioned in this paper would be designated microcystins today) were developed by Kfir *et al.* [47]. In the year 2000, McElhiney, Lawton and Porter showed the production of recombinant antibodies against microcystins from a naïve library (obtained from non-immunized animals), however, of relatively low affinity [48]. Two years later, the same group published another attempt to produce recombinant single-chain antibodies against microcystin-LR from another library [49]. The affinity was improved, but surprisingly, the most affine clone preferred microcystin-RR. Recently, it was shown by Drake *et al.* that antibodies against microcystins can be produced in plants [50]. However, the yield was only 0.006% of leaf fresh weight. In 2001, Zeck *et al.* reported a monoclonal antibody targeted against a partial structure of microcystins and nodularins, the rare amino acid Adda [37]. This approach resolved the problem of unknown microcystins and their respective cross-reactivities in a quite general way. They could show that all tested microcystins displayed very even cross-reactivities, leading to a true sum value, something which cannot be achieved with instrumental methods, such as LC-MS/MS, since the retention times and masses of unknown toxins are *per se* not known. The detection limit of microcystin LR was calculated to be 0.068 μg/L. Some applications of this antibody were reported in the literature [19,51–56]. Shortly later, Fischer *et al.* presented a similar concept based on sheep antibodies with nearly the same sensitivity [36]. In the year 2009, a new monoclonal antibody of very broad selectivity was developed [57]. Although the authors used a microcystin-LR derivative as an immunogen, they could identify a clone of broad selectivity by a competitive test protocol with different microcystins. Even Adda derivatives are bound by this antibody.

Several immunoassays against microcystins based on monoclonal antibodies have been developed by Saito *et al.* [58] and Nagata *et al.* [59–65]. Remarkable was the development of anti-idotype monoclonal antibodies [63–65], which enabled the set-up of sandwich-type immunoassays, which are otherwise very difficult for small analytes, such as cyanotoxins. A similar concept based on polyclonal antibodies was presented by Liu *et al.* in 1996 [66]. Nagata *et al.* also showed some environmental screening and monitoring applications [60], as well as the detection of microcystins by immunohistochemistry [67]. It could be shown that although the clone M8H5 was developed on the base of microcystin LR, the cross-reactivities were quite broad [68], which allows the semiquantitative detection of most microcystins. In addition, an immunoaffinity enrichment of microcystins from water was presented [69].

In the year 2001, Zeck *et al.* isolated a monoclonal antibody against all microcystins containing an arginine (*R*) at position 4 [70], which include the frequently occurring variants microcystin-LR and microcystin-RR. This antibody was used in quite a few applications [52,71–98]. Mikhailov *et al.* presented highly selective, monoclonal antibodies against nodularin [99] with negligible reactivity against microcystins.

In 2006, Haskard published a detailed report [100] about microcystin immunoassays based on the clone AD4G2. This clone is recommended, if a full coverage of all microcystins, nodularins and their derivatives is required. Recently, the government of Nova Scotia (Canada) commissioned a comparison of two test kits for the measurement of microcystin concentrations [101]. In this context, it has to be mentioned that according to the Guidelines for the Canadian Drinking Water Quality, a concentration of 1.5 μg/L of microcystin LR is allowable [102]. A new monoclonal antibody (clone MC8C10 or 8C10) preferentially against the variant microcystin-LR was presented in 2007 [103–105].

In general, it can be stated that only for microcystin and related toxins a sufficiently characterized antibody repertoire is available (Table 3). For all other toxins the development and thorough examination of high quality antibodies would be highly desirable. This situation delays the development of multianalyte immunoassays and biosensors in this field, although the general approach and technology is already known [106–113] and some systems are nowadays even commercially available (e.g.: http://www.randox.com/, http://www.saw-instruments.com/, http://www.biacore.com/, http://www.concentris.ch). In addition, it can be observed that many (re)sellers of antibodies neither deliver a traceable clone number for identification, nor do they have a minimum of antibody characteristics or citations of scientific work available. Since studies with such materials cannot be considered to be reproducible and hence not “scientific” in a fundamental sense, their use is highly discouraged. Unfortunately, antibodies (and this includes the recombinant variants) are still very expensive reagents. All efforts to reduce the cost of antibodies have not changed the situation significantly. Therefore, cost might be still the most severe limitation of the application of antibodies in analytical protocols.

## 3. Enzyme Inhibition

Cyanobacterial toxins often attack specific biochemical targets in other species. This selectivity enables the use of these targets for analytical purposes. The most well-known entities are protein phosphatases, which are very important enzymes to control the phosphorylation status in a cell. In mammals, microcystins mainly affect the liver, which finally disintegrates and leads to the death of the individual. On a molecular level, different protein phosphatases are inhibited, for example protein phosphatase 1 (PP1), protein phosphatase 2A (PP2A) and to a lesser extent protein phosphatase 2B (PP2B) [114]. Okadaic acid is an inhibitor of PP2A and at higher concentration, also of PP1.

If the respective enzyme can be obtained commercially or isolated, it can be advantageously used for very selective and sensitive enzyme inhibition tests. They can either be performed in a traditional format based on the measurement of the enzyme activity; in this case they act as a phosphatase on a—most often artificial—substrate. Another option is the use of the enzyme as a pure binder, which can be used in an immunoassay- or immunosensor-like format. Some of the enzymes are not only isolated from natural sources, but are produced by recombinant techniques. This opens up the opportunity for structural modifications, which might improve the applicability of the enzymes in biosensing and other fields.

A very early application of protein phosphatase inhibition was presented by the group of Holmes in 1993 [115,116]. By this technique, they could detect okadaic acid and microcystins in one system. It has to be noted that this is also an early example of effect-related analysis [18], what Holmes termed *liquid chromatography-linked protein phosphatase bioassay*. A comparison of LC-MS, ELISA and phosphatase assay was published in 2001 [117]. Also the problem of unknown microcystins was discussed in the paper.

No antibodies against anatoxin-a(s) seem to be available. However, the neurotoxin anatoxin-a(s) is known to be an acetylcholine esterase (AChE) inhibitor. Therefore, Devic *et al.* engineered AChE mutants based on the enzyme from the insect *Drosophila*. The inhibition rates of anatoxin-a(s) were much higher than those of organophosphorous pesticides, such as paraoxon, malaoxon, dichlorvos, carbaryl and others [118]. The limit of detection was found to be 0.5 nmol/L for a screen-printed amperometric sensor. Villatte *et al.* showed an enzyme inhibition biosensor based on AChE from electric eel, and achieved a detection limit of 1 μg/L of anatoxin-a(s) [119].

## 4. Aptamers

Aptamers, which are small DNA or RNA oligomers, which have been fished by several rounds of enrichment out of large random libraries, can be produced against nearly any analyte. Nevertheless, there seem to be some restrictions, which might be caused by the very limited chemical variability of the monomers. It can be assumed that aptamers are most useful for the binding of complex analytes, such as proteins. Many cyanobacterial toxins are of medium size, which makes them at least theoretically accessible to the selective binding of aptamers. An aptamer against microcystin-LR with limited affinity was reported in 2004 (http://www.ncbi.nlm.nih.gov/pubmed/15569506) and used recently for an electrochemical biosensor [120]. However, it is puzzling that a binder of low affinity should lead to a sensor of extreme sensitivity. Another group reported the selection of DNA aptamers of higher affinity [121] in 2012. They also used an electrochemical setup for the design of a biosensor system and claimed a detection limit of 10 pM. Recently, an aptamer against saxitoxins (STX) was reported [122]. However, an affinity constant was not determined, yet. Another group [123] also selected an aptamer (“F3”) to saxitoxin, which displayed an affinity of about 10^7^ L/mol (100 nM).

## 5. Molecularly Imprinted Polymers (MIPS)

Molecularly imprinted polymers (MIPS) are completely artificial binders based on synthetic monomers. More than 2,200 articles and nearly 100 review articles have been written until today (Web of Science: Title = (molec* and imprint* and polym*; July 8, 2013). Therefore, it is not surprising that even a “review of reviews” was published recently [124]. Briefly, a template molecule (analyte) is mixed with one or several functional monomers and a porogen (solvent) and a polymerization catalyst or starter. In most cases a high concentration of a cross-linker (multivalent monomer) is also added. After the polymerization is finished (e.g. by heating), the material is ground and extracted thoroughly to remove most of the template molecules. The remaining molecular cavities can be used for selective binding of the respective analyte. The most striking advantages are robustness and often cost. The imprinted polymer might be regenerated nearly indefinitely without loss of “activity”. Depending on the monomer and cross-linker types, the polymer can be treated with very harsh cleaning solutions, to get rid of even very sticky contaminations. Furthermore, the use of organic solvent extracts or acidic, basic or other aggressive solutions is suitable for MIPs, in contrast to protein-based binder, which would be denatured [125]. If the analyte is readily available and relatively cheap, the MIPs are also of very low cost in contrast to antibodies. On the other hand, MIPs also have severe drawbacks, which have limited their application in many fields. MIPs are the complement to polyclonal antibodies, not to monoclonal ones, since there is a broad distribution of affinities and selectivities of the binding sites. Even worse is the fact that each synthesis batch is somewhat different, and a specific binder can be hardly reproduced. In addition, in contrast to other binding materials, the capacity of MIPs often is quite low. Furthermore, the relatively hydrophobic polymer leads to high non-specific binding, which requires very careful optimization of binding and elution conditions. In addition, the use of MIPs for highly aqueous samples is still difficult. Due to the relatively non-flexible structure of the polymer, either the binding and elution kinetics is poor, or the capacity is low, if only surface binding is allowed. Since MIPs can be considered to be bio-inspired or bio-mimicking materials, the respective sensors, will be designated biosensors in this context.

## 6. Biosensors

### 6.1. Optical Biosensors

In 1999, the group of Marquette *et al.* published a chemiluminescence immunosensor for the detection of okadaic acid with a limit of detection of 2 μg/kg mussel homogenate [126]. The group of Moreno-Bondi recently reported the use of a “Leopard Array Biosensor”, the commercial version of the Naval Research Laboratory (NRL, Washington, DC, USA) Array Biosensor [108,127]. This paper [128] describes a system for the parallel detection (six samples) of microcystins in freshwater in 60 min. The detection limit was around 0.016 μg/L.

Recently, an interesting immunosensor was published, which used the rapid fluorescence quenching of a microcystin-DNA-fluorescein conjugate by the interaction with graphene [129]. The detection limit was determined to be 0.14 μg/L, which is sufficient to meet the requirement of the provisional WHO limit for drinking water [130]. However, from the calibration curves, a detection limit > 1 μg/L would be more realistic. In addition, the shown microcystin labeling chemistry seems to be quite uncommon, since the arginine side chain is usually considered to be nonreactive. Most likely, the thiol group of the oligonucleotide directly reacted with the beta-alanine of the microcystin. The structure of the conjugate would be different; nevertheless, the mechanism of the sensor would not be influenced considerably. Unfortunately, the potential quenching effect of matrix compounds, such as humic acids was not examined. Another paper showed the application of graphene for an optical biosensor [131]. Gold nanoparticles were used to quench the photoluminescence of graphene oxide. The limit of detection was determined to be 0.5 μg/L. This system also seems to need further characterization and validation.

**Figure 1.**
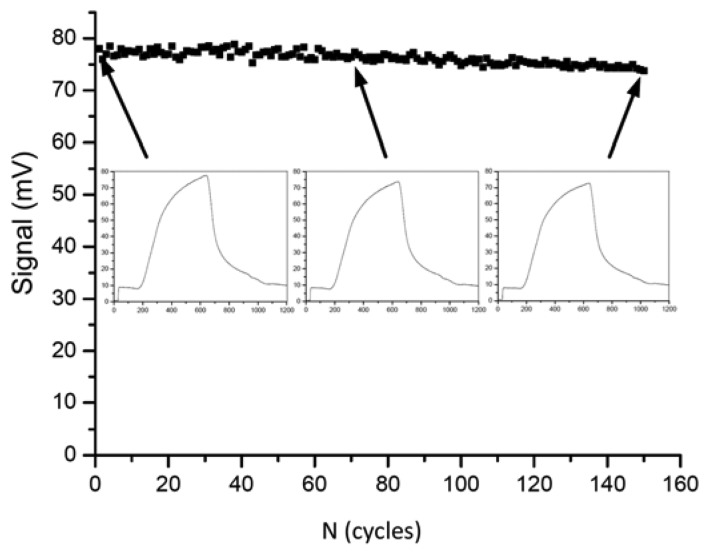
One hundred and fifty repetitive sensor cycles in absence of microcystin. Reprinted with permission from [132] Copyright 2009 Elsevier.

In the year 2009, Long *et al.* presented a fiber-optical biosensor for the highly reproducible detection of microcystins [132]. The limit of detection was given as 0.03 μg/L and the measuring range was 0.1–10 μg/L. An impressive series of 150 regeneration cycles was shown with less than 5% of activity loss (Figure 1).

A surface-plasmon resonance-(SPR)-based biosensor for microcystins was presented by Herranz *et al.* in 2010 [81]. The authors compared four monoclonal antibodies in different formats, from which only one clone showed a sufficient and selective signal. An amino-terminated self-assembled monolayer (SAM) with covalently immobilized microcystin-LR was found to be optimal. More than 40 regeneration cycles (50 mM NaOH) could be shown. The detection limit was determined to be 0.073 μg/L, well below the WHO level. Tap water was tested, but more complex samples have not been examined, yet.

A biosensor based on a conventional SPR system was presented [133] in 2009. Fifty assay cycles could be shown and a limit of detection of around 1 μg/L could be achieved. One assay needed about 50 min. A much faster SPR assay was presented by Yakes *et al.* in 2011 [134]. They accelerated their system for saxitoxins (STX) detection to less than 5 min including the regeneration step by optimization of all incubation steps. However, the limit of detection (LOD) was only around 1 μg/L of STX. Other toxins showed only a negligible inhibition effect at 300 mg/L. Unfortuntately, not much information was given about the antibody.

**Figure 2.**
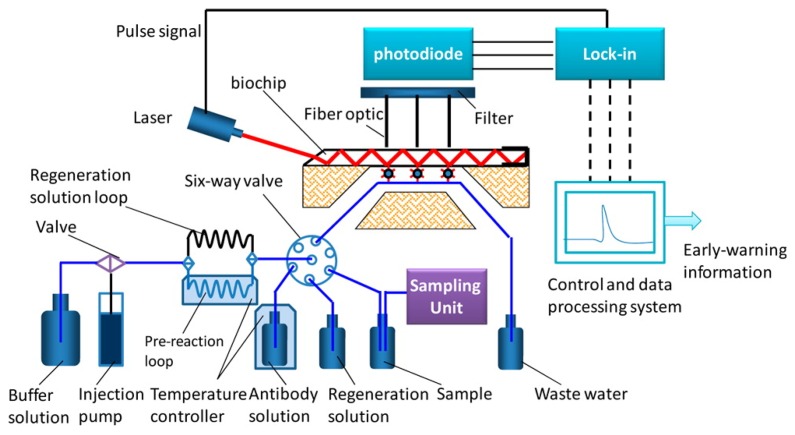
Modified scheme of the Automated Online Optical Biosensing System (AOBS) of Shi *et al.*, reprinted with permission from [135]. Copyright 2013 American Chemical Society.

**Figure 3.**
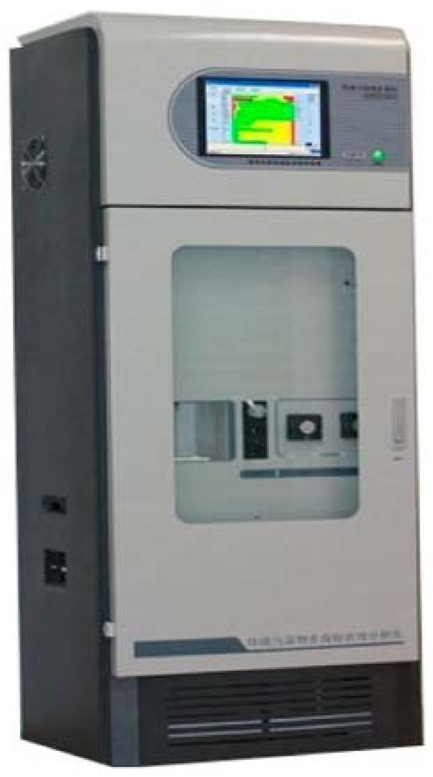
Photograph of the AOBS of Shi *et al.* modified. Reprinted with permission from [135]. Copyright 2013 American Chemical Society.

In 2013, an automated online biosensing system for the detection of microcystin-LR was published [135], which was called automated online optical biosensing system (AOBS). It seems to have some similarity to the Total Internal Reflection Fluorescence (TIRF) systems published by the group of Gauglitz [136]. The limit of detection of the AOBS was reported to be 0.09 μg/L. The scheme and a photograph of this recent system are depicted in Figures 2 and 3.

**Figure 4.**
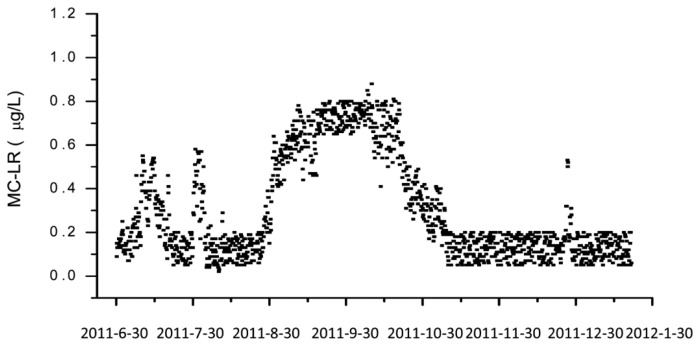
Long-term measurements of microcystin-LR in Lake Tai, China. Reprinted with permission from [135]. Copyright 2013 American Chemical Society.

However, the most remarkable point of this paper is the documentation of a long-term application of this automated system over a time frame of nearly one year at Lake Tai, one of the largest freshwater lakes in China (Figure 4). A measurement was performed each six hours, including one calibration run per day. The biochip had to be exchanged every month. The reagent solutions needed to be refilled every two weeks.

### 6.2. Mass-Dependent Biosensors

About 10 years ago, a piezoelectric system for the detection of microcystins was presented [137]. The sensing layer consisted of a MIP particle coating on a piezoelectric crystal. The response time was in the range of 5–10 min, the detection limit was around 35 nM (≈ 35 μg/L). Considering the WHO value of 1 μg/L for drinking water, it was clear that the sensor was not sensitive enough. Therefore, a preconcentration with MIP cartridges was added, which improved the sensitivity considerably. The minimum detectable amount of toxin was 0.35 μg/L for the combined system, however, a manual enrichment of the samples was required.

Bergantin and Sevilla presented one of the rare setups based on biological receptors [138]. They used sodium channel receptors from the electric eel (*Electrophorus electricus*), which were immobilized on a quartz crystal microbalance. The lowest concentration examined was 100 μg/L of saxitoxin. In the year 2011, a study was published, in which cantilever biosensors were used for the detection of microcystin-LR [139]. Although the sensor was quite sensitive with a detection limit around 0.25 ng/L of microcystin-LR, the signal change was highly dependent on the matrix, which makes the practical application quite difficult. In addition, the authors showed that a combination of a monoclonal with a polyclonal antibody seemed to work as a sandwich immunoassay, which is quite uncommon for such a small analyte. Unfortunately, the identity of antibodies used could not be established.

Recently, a system based on a quartz microbalance (QCM) was presented [140]. Without any amplification, the biosensor showed a quite poor sensitivity; however, after a double amplification with a dendritic surfactant forming a liposome array and gold nanoparticles a detection limit around 1 μg/L was achieved. The protocol needed more than three hours for a measurement, which was finally performed in air.

Fournel *et al.* reported the detection of okadaic acid by a love wave biosensor system [141,142]. Since okadaic acid is a relatively large molecule, the authors claim to have performed a sandwich immunoassay based on a single polyclonal serum. However, some validation experiments (e.g., real samples) of this acoustic wave system are lacking. A direct detection of okadaic acid seems to be impossible. Particularly interesting is the design of the microfluidic chamber, which was found by a flow simulation.

### 6.3. Electrochemical Biosensors

A label-free capacitive immunosensor with silver particles was published in 2008 [86]. The system was described to be extremely sensitive with a detection limit of 7 pg/L of microcystin-LR and a linear range between 10 pg/L and 1 μg/L. Repeated regeneration (up to 43 times) was possible. Unfortunately, the used reference method (HPLC) had a detection limit, which is 10^8^ times higher, and therefore, the claimed sensitivity has to be considered as non-confirmed.

In 2010, a paper was published showing the use of carbon nanohorns for the setup of an electrochemical immunosensor for microcystin-LR [143]. The detection limit was calculated to be at 0.03 μg/L. Unfortunately, neither the antibody nor the performance in real samples was characterized in detail, which also means that validation for this system is still lacking.

Campàs *et al.* showed a proof-of-concept study that enzyme amplification can lead to significant improvement of sensitivity [144]. A biosensor based on inhibition of protein phosphatase 2A (PP2A) could be sensitized by more than four orders of magnitude by use of diaphorase/NADH oxidase amplification step. However, considering the experience with amplification in immunoassays, problems with reproducibility might be a disadvantage.

In 2011 a graphene-based, electrochemical immunosensor was published, which used a PtRu alloy for signal amplification [145]. A very low detection limit of about 0.01 μg/L of microcystin-LR was claimed. However, the mechanism of the sensor is somewhat obscure, since the authors used a sandwich-type immunoassay with no further information about the antibody pair, which is the most interesting part of the sensor. In addition, no cross-reactivities or matrix effects have been tested.

Recently, an extremely sensitive electrochemical immunosensor against microcystin-LR was presented [146]. The authors claimed a detection limit of 3.7 × 10^−17^ M or 37 fg/L of microcystin-LR, which is about one million times more sensitive than any other biosensor in this field. However, it is difficult to explain, how the authors obtained antibodies of such an extraordinary affinity by a standard method. Otherwise no microcystin binding could be expected due to thermodynamic reasons. Therefore, this paper might need some independent confirmation.

Another electrochemical system based on graphene in combination with peroxidase/carbon nanospheres was also published in 2013 [147]. The detection limit of 0.016 μg/L microcystin seems to be more realistic. Real samples and interferences have been examined.

### 6.4. Other Biosensors

Even Nuclear Magnetic Resonance (NMR) has been used for the construction of a microcystin immunosensor [148]. The aggregation of magnetic nanoparticles was influenced by the addition of the sample as competitor. The detection limit was defined to be 0.6 μg/L. Although the protocol was relatively simple, an incubation time of 3 h at 37 °C was needed. Surprisingly, the calibration curve (supplementary material) was highly linear between 1 μg/L and 20 μg/L, which is hardly the case in such competitive assays. Furthermore, it is possible to identify a cyanobacterial hazard indirectly, by the detection of cyanobacterial DNA or RNA (e.g., [149]). However, this approach will not be covered by this review.

### 6.5. Multiplexed Biosensors/Biosensor Arrays

In 2009, a rapid chemiluminescence biosensor was published by Lindner *et al.* [73]. The multichannel immunosensor is based on a capillary ELISA technique, which needs about 18 min for a measurement. A detection limit of 0.2 μg/L was reached, which is suitable to monitor the maximum concentration for microcystin-LR proposed by the WHO. The setup was designed for a detection of up to three toxins, however the measurements are performed successively. The analysis of microcystin-LR and Staphylococcal Enterotoxin B (SEB) was shown. Apparently, regeneration was not considered.

**Figure 5.**
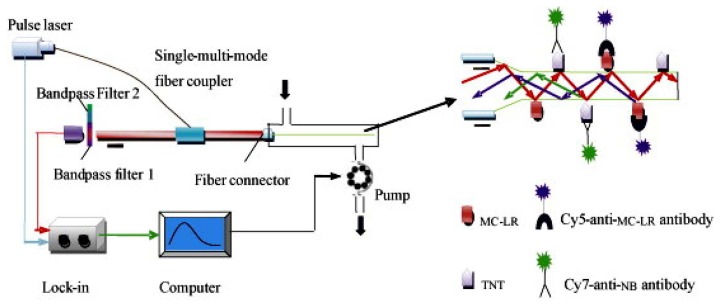
Setup of a fiber-optical immunosensor for the parallel detection of microcystin-LR and TNT. Reprinted with permission from [104]. Copyright (2010) Elsevier.

Although, the development of fiber-optic biosensors has been presented quite a time ago [150–152], the use for multianalyte detection was not frequently shown [153–157]. Long *et al.* published a paper describing fiber-optic biosensors (see Figure 5) for the detection of multiple analytes, such as microcystin-LR and TNT [104].

**Figure 6.**
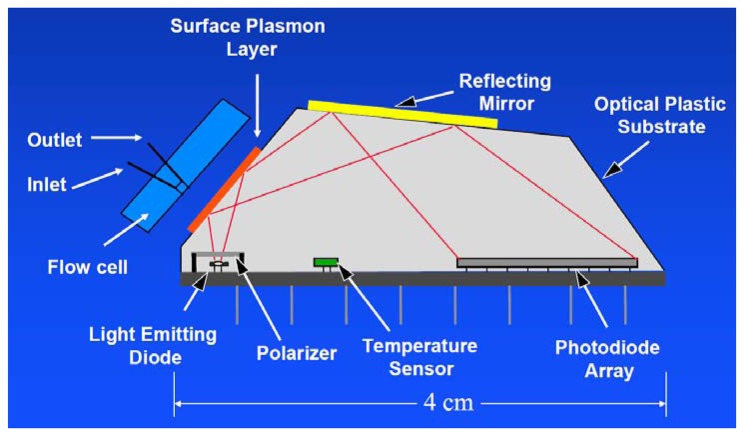
Miniature Surface-Plasmon Resonance (SPR) Sensor Platform (modified). Reprinted with kind permission of C. E. Furlong, Seattle.

**Figure 7.**
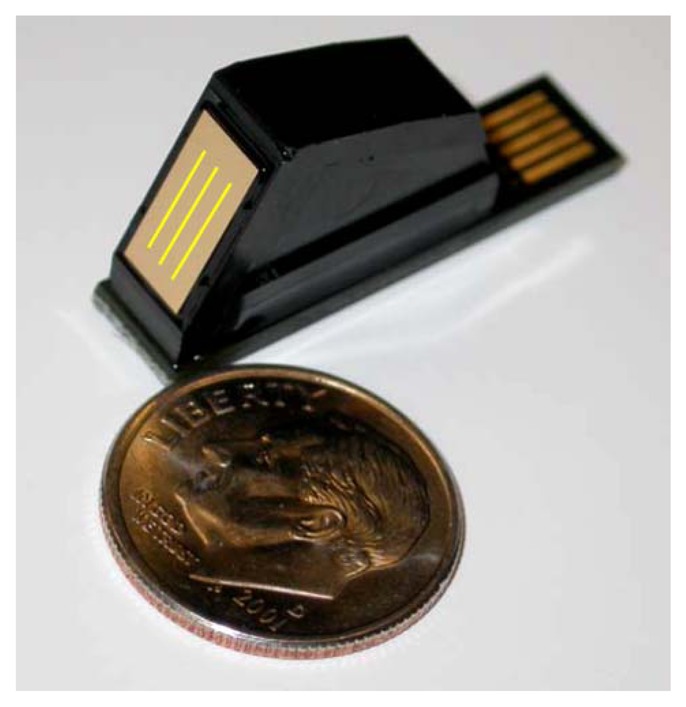
Spreeta 2000 sensor chip. Reprinted with kind permission of C. E. Furlong, Seattle.

A mobile surface-plasmon resonance (SPR) biosensor for the detection of domoic acid was published by Stevens *et al.* in 2007 [158]. Their six-channel system contained two three-channel Texas Instruments Spreeta 2000 sensor chips [159], which is an integrated device, useful for the construction of compact SPR setups (Figures 6 and 7). The authors produced their own domoic acid antibodies in rabbits, which were affinity purified on a different carrier conjugate. More than 50 regeneration cycles were possible according to a statement in the paper. A calibration curve between 3 μg/L and 600 μg/L of domoic acid (in PBS) was shown. Measurements in clam extracts were also possible.

**Figure 8.**
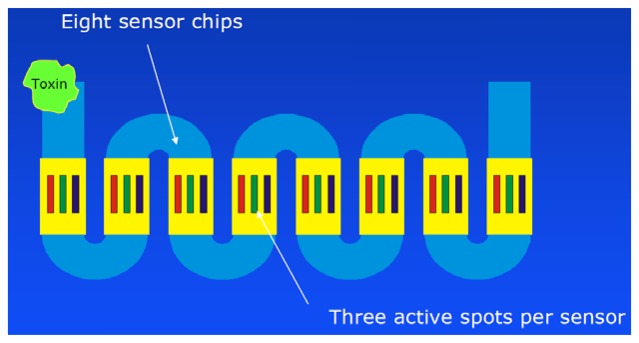
Scheme of the 24-channel SPR sensor based on 3-channel Spreeta chips (modified). Reprinted with kind permission of C. E. Furlong, Seattle.

In addition, a portable 24-analyte SPR instrument (Scheme shown in Figure 8) was presented [160]. A similar system (12 channels) is now commercialized by Seattle Sensor Systems (http://seattlesensors.com).

A bead-based, multiplexed system for the detection of microcystin-LR and benzo[a]pyrene was shown recently by Yu *et al.* [161]. Quantum dots of two different emission wavelengths have been used as labels. One assay cycle could be performed in 30 min. Although sensitivity advantages and better reproducibility are claimed for quantum dot assays, this could not be shown, yet. It has to be kept in mind that competitive immunoassays due to their inhibition mechanism usually do not profit significantly from improved labels, which was recently shown in a comparison of the usual colorimetric detection with highly sensitive ICP-MS [162].

In 2012 the group of Marquette published a multiplexed biosensor based on colorimetric staining of microarrays [163]. They tried to determine okadaic acid, atrazine, 2,4-D, TNT and RDX in parallel. Unfortunately, they could only partially control the mutual influence of the assays on one chip—a problem, which was discussed previously [107]. This confirms the notion that indirect hapten arrays are less suitable for the parallel analysis of single analytes and hence should be preferentially used for group selective assays with carefully selected (most often) monoclonal antibodies [164,165]. Very recently, Szkola *et al.* presented a microarray biosensor for the detection of phycotoxins (http://en.wikipedia.org/wiki/Phycotoxin) in shellfish extracts [166]. The system is based on a chemiluminescence biosensor first developed for the monitoring of water [106], which was then extended to the application of allergy diagnosis [113], fast detection of antibiotics in raw milk [112] and honey [167]. The new study showed the detection of saxitoxin, domoic acid and okadaic acid. At least 25 measurement cycles could be demonstrated. However, some corrections had to be performed to compensate for some regeneration losses. The total assay time without sample extraction was 20 min. Different dilution factors are necessary for different toxins, which makes it difficult to detect several toxins in parallel.

## 7. Conclusions/Outlook

The detection of cyanobacterial and other algal toxins is an active area of research. Due to the complex structure of the analytes, the use of biosensors is particularly attractive. Considerable efforts have been made to develop sensitive, selective, and fast biosensors for these applications. However, only very few systems already overcame the lab status. Particularly, the development of multianalyte systems seems to be limited by the lack of high-quality antibodies for most of the toxins. Antibodies are by far the most frequently used reagents. It could be shown that some immunoassays and commercial antibodies are on the market, which are obviously useful for sensitive cyanotoxins analysis. Unfortunately, some assays, biosensors and most antibodies lack sufficient characterization. If the prospective customers are not prepared to do the lacking validation work in their own lab, these products should be avoided, since the results cannot be properly interpreted due to unknown cross-reactivities and other issues. The prospective user should always ask for full antibody/assay characterization data before purchase (The tables presented in this paper are neither complete nor definite and should be only used for a first orientation). In many applied studies the authors are not aware of these pitfalls. Often, general statements, such as “immunoassays/antibodies display a higher …” or “biosensors are less …” show that some users assume that general statements about the performance of e.g., “microcystin immunoassays” are possible. However, similar to nearly all products of everyday life, there are good and bad ones. A test about one antibody or immunoassay or biosensor usually cannot be transferred to another. Exactly this is sometimes done by using one immunoassay and citing and discussing another. A special problem is the objective evaluation of immunosensors. Some researchers disregard that one of the major factors defining the performance of an immunosensor is the used antibody, which highly influences the sensitivity of the sensor by the affinity of the reagent and the selectivity by its cross-reactivity properties. The majority of the biosensor papers mentioned in this review do not give sufficient information about these critical reagents, which makes it disputable to compare the systems directly in terms of performance.

Neither aptamers, nor MIPs are competitive with antibody-based binders at the moment. In contrast, biosensors using protein phosphatases or biological receptors are already useful tools for the purpose of algal toxin detection—obviously only for these subclasses of toxins, which specifically inhibit these enzymes or bind to the respective receptors. Biosensors based on enzyme inhibition or receptor affinity can be considered to be systems for effect- or toxicity-related analysis [18,168]. This is a more comprehensive approach, in comparison to the conventional instrumental analysis, which may lead to false negatives in cyanotoxin analysis, e.g., due to lack of standard and calibration materials. Similar problems can even occur with antibody-based biosensors, if the antibody cross-reactivity pattern is not broad enough or even unknown.

## Figures and Tables

**Table 1.** Commercial immunoassays against microcystins and nodularins.

**Company**	**Designation**	**Limit of Detection** *	**CR** *	**Ref.**
Abraxis	Microcystins, ELISA Kit, Coated Tube, PAb, Rabbit	0.15 μg/L Microcystin LR	+	–
Abraxis	Microcystins Field Screen, ELISA Kit, PAb	0.29 μg/L Microcystin LR	+	–
Abraxis	Microcystins/Nodularins (ADDA), ELISA Kit, PAb, Sheep	0.1 μg/L Microcystin LR	+	[36]
Abraxis	Microcystins/Nodularins (ADDA) ES, ELISA Kit, PAb, Sheep	0.04 μg/L Microcystin LR 1	+	[36]
Abraxis	Microcystins/Nodularins-DM, ELISA Kit, MAb AD4G2, Mouse	0.1 μg/L Microcystin LR	+	[37]
Abraxis	Microcystins ELISA for Serum (MTP), MAb AD4G2, Mouse	0.4 μg/L Microcystin LR	+	[37]
Abraxis	Microcystins Strip Test, MAb AD4G2, Mouse	1 μg/L Microcystin LR	+	[37]
Beacon	Microcystin Tube Kit, PAb, Rabbit	0.3 μg/L Microcystin LR	+	–
Beacon	Microcystin Plate Kit, PAb, Rabbit	0.1 μg/L Microcystin LR	+	–
Beacon	Nodularin Plate Kit, PAb, Rabbit	0.4 μg/L Nodularin	–	–
Envirologix	QuantiPlate™ Kit for Microcystins, MTP	0.18 μg/L / 0.03 μg/L	+	–
Envirologix	QualiTube™ Kit for Microcystins, Tubes	0.3 μg/L Microcystin	+	–
Enzo	Microcystins (Adda specific) ELISA kit, PAb, Sheep	0.1 μg/L Microcystin LR	+	[36]
Modern Water	EnviroGard™ Microcystins Plate	0.1 μg/L Microcystin	+	–
Taiwan Algal Service	Microcystins Plate Kit	0.1 μg/L Microcystin	–	–
ZEU Immunotec	Microcystest™ Kit 2	0.2 μg/L Microcystin	–	–
ZEU Immunotec	Microcystest™ Tube 2	0.2 μg/L Microcystin	–	–

Notes:

* or lowest standard solution, data from manufacturer. CR: cross-reactivity data available form manufacturer. PAb: Polyclonal Antibody, MAb: Monoclonal Antibody, ELISA: Enzyme-linked immunosorbent assay, MTP: Microtiter plate, ES: Enhanced sensitivity, DM: direct monoclonal, Adda: [(2*S*,3*S*,8*S*,9*S*)-3-amino-9-methoxy-2,6,8-trimethyl-10-phenyldeca-4,6-dienoic acid];

1 additional preincubation and washing step required;

2 Phosphatase inhibiton tests.

**Table 2.** Commercial immunoassays against other cyanobacterial toxins and shellfish toxins ^†^.

**Company**	**Designation**	**Limit of detection** *	**CR**	**Ref.**
Abraxis	Anatoxin-a ^1^	10 μg/L Anatoxin-a	–	–
Abraxis	Brevetoxin ^2^ (NSP), ELISA Kit	0.05 μg/L Brevetoxin	+	–
Abraxis	Domoic Acid ^3^ (ASP), ELISA Kit	Ca. 0.01 μg/L Domoic Acid	+	[45]
Abraxis	β-N-Methylamino-L-alanine ^4^	4 μg/L BMAA	+	–
Abraxis	Cylindrospermopsin ELISA	0.04 μg/L Cylindrospermopsin	+	-
Abraxis	Okadaic Acid ^5^ (DSP), ELISA Kit	ca. 1 μg/L Okadaic Acid	+	–
Abraxis	Saxitoxin	0.015 μg/L Saxitoxin	+	–
AntiProt	Anti-saxitoxin ELISA-Stick	1 ng Saxitoxin	–	–
Beacon	Brevetoxin Plate Kit	0.1 μg/L Brevetoxin	–	–
Beacon	Cylindrospermopsin Plate Kit	0.1 μg/L Cylindrospermopsin	+	–
Beacon	Okadaic Acid Plate Kit	0.2 μg/L Okadaic Acid	–	–
Beacon	Saxitoxin	0.02 μg/L Saxitoxin	+	–
Beacon	Neo-Saxitoxin ^6^	0.03 μg/L Saxitoxin	+	–
Biosense Laboratories	Domoic Acid (ASP) ELISA kit	ca. 0.01 μg/L Domoic Acid	+	[45]
r-biopharm	Ridascreen Fast PSP SC	50 mg/L Saxitoxin	+	–
Taiwan Algal Science	Domoic Acid (ASP) ELISA kit	0.05 μg/L Domoic Acid	–	–
ZEU Immunotec	Okatest2	44 μg/kg Okadaic Acid	–	[46]

Note:

† Not necessarily of cyanobacterial origin;

* or lowest standard solution, data from manufacturer;

1 Receptor Binding Assay based on nicotinic acetylcholine receptors;

2 http://en.wikipedia.org/wiki/Brevetoxin;

3 http://en.wikipedia.org/wiki/Domoic_acid;

4 http://en.wikipedia.org/wiki/Beta-Methylamino-L-alanine;

5 http://en.wikipedia.org/wiki/Okadaic_acid;

6 http://en.wikipedia.org/wiki/Neosaxitoxin; CR: cross-reactivity data available.

**Table 3.** Commercial antibodies against cyanobacterial and some marine toxins ^†^.

**Analyte(s)**	**Company (short)**	**Antibody, Clone(s)**	**Ref.**
Microcystin/Adda	Mybiosource	Mouse, 6D583 ^1^	[37]
Microcystin LR	Mybiosource	Mouse, B762M, B764M	–
Microcystin LR	Hytest	Mouse, C64A1	–
Microcystin LR	Hytest	Mouse, C64C12	–
Microcystin LR	Abnova	Mouse, C64A1	–
Microcystin LR	Abnova	Mouse, C64C12	–
Microcystin LR	Abcam	Mouse, C64A1	–
Microcystin LR	Abcam	Mouse, C64C12	–
Microcystin LR	US Biological	Mouse, 10J35 ^2^, 10J36 ^2^, 6D584 ^2^	[70]
Microcystin LR	Antibodies online	Mouse, B762M, B764M	–
Microcystin LR	Biorbyt	Mouse, B762M, B764M	–
Microcystin LR	Acris	Mouse, B762M, B764M	–
Microcystin LR	Enzo	Mouse, MC10E7	[70]
Microcystin/Adda	Enzo	Mouse, AD4G2	[37]
Microcystin LR	Meridian	Mouse, B762M	–
Microcystin LR	Meridian	Mouse, B764M	–
Cylindrospermopsin	Meridian	Mouse, B759M, B761M	–
Domoic Acid	Meridian	Rabbit, Pab	–
Domoic Acid	Acris	Rabbit, Pab	–
Okadaic Acid	Meridian	Rabbit, Pab	–
Okadaic Acid	LSBio	Mouse, 7E1	–
Okadaic Acid	Santa Cruz	Mouse, 7E1	+
Okadaic Acid	LSBio	Mouse, 70	–
Saxitoxin	Agrisera	Rabbit, Pab	–
Yessotoxin ^3^	Meridian	Rabbit, Pab	–

Note:

† Not necessarily of cyanobacterial origin;

1 Probably identical to AD4G2;

2 Probably identical to MC10E7;

3 http://en.wikipedia.org/wiki/Yessotoxin, PAb: Polyclonal antibody.
